# Expression Patterns of MiR-125a and MiR-223 and Their Association with Diabetes Mellitus and Survival in Patients with Non-ST-Segment Elevation Acute Coronary Syndrome

**DOI:** 10.3390/biomedicines11041118

**Published:** 2023-04-07

**Authors:** Gloria M. Gager, Ceren Eyileten, Marek Postuła, Anna Nowak, Aleksandra Gąsecka, Bernd Jilma, Jolanta M. Siller-Matula

**Affiliations:** 1 Division of Cardiology, Department of Internal Medicine II, Medical University of Vienna, 1090 Vienna, Austria; gloria.gager@meduniwien.ac.at; 2 Department of Clinical Pharmacology, Medical University of Vienna, 1090 Vienna, Austria; 3 Centre for Preclinical Research and Technology (CEPT), Department of Experimental and Clinical Pharmacology, Medical University of Warsaw, 02-091 Warsaw, Poland; 4 Genomics Core Facility, Center of New Technologies (CeNT), University of Warsaw, 00-927 Warsaw, Poland; 5 Doctoral School, Medical University of Warsaw, 02-091 Warsaw, Poland; 6 Department of Cardiology, Medical University of Warsaw, 02-091 Warsaw, Poland

**Keywords:** acute coronary syndrome, NSTEMI, NSTE-ACS, microRNA, miRNA, miR-125a, miR-125b, miR-223, long-term all-cause mortality

## Abstract

**Background**: MicroRNAs (miRNA, miR) are small, non-coding RNAs which have become increasingly relevant as diagnostic and prognostic biomarkers. The objective of this study was the investigation of blood-derived miRNAs and their link to long-term all-cause mortality in patients who suffered from non-ST-segment elevation acute coronary syndrome (NSTE-ACS). **Methods**: This study was an observational prospective study, which included 109 patients with NSTE-ACS. Analysis of the expression of miR-125a and miR-223 was conducted by polymerase chain reaction (PCR). The follow-up period comprised a median of 7.5 years. Long-term all-cause mortality was considered as the primary endpoint. Adjusted Cox-regression analysis was performed for prediction of events. **Results**: Increased expression of miR-223 (>7.1) at the time point of the event was related to improved long-term all-cause survival (adjusted (adj.) hazard ratio (HR) = 0.09, 95% confidence interval (95%CI): 0.01–0.75; *p* = 0.026). The receiver operating characteristic (ROC) analysis provided sufficient c-statistics (area under the curve (AUC) = 0.73, 95%CI: 0.58–0.86; *p* = 0.034; negative predictive value of 98%) for miR-223 to predict long-term all-cause survival. The Kaplan–Meier time to event analysis showed a separation of the survival curves between the groups at an early stage (log rank *p* = 0.015). Higher plasma miR-125a levels were found in patients with diabetes mellitus vs. in those without (*p* = 0.010). Furthermore, increased miR-125a expression was associated with an elevated HbA1c concentration. **Conclusions**: In this hypothesis-generating study, higher values of miR-223 were related to improved long-term survival in patients after NSTE-ACS. Larger studies are required in order to evaluate whether miR-223 can be used as a suitable predictor for long-term all-cause mortality.

## 1. Introduction

Acute coronary syndrome (ACS) is the leading cause of mortality and morbidity, with an increasingly observed occurrence of non-ST-segment elevation myocardial infarction (NSTEMI) [1,2]. Under the expression *ACS,* three manifestations of the disease are summarized, namely, ST-segment elevation myocardial infarction (STEMI), non-ST-segment myocardial infarction (NSTEMI) and unstable angina (UA). However, non-ST-segment acute coronary syndrome (NSTE-ACS) comprises the conditions NSTEMI and UA [3].

Regarding appropriate risk stratification during and after the event, biomarkers have an undeniably important role—at the forefront is cardiac troponin T (cTnT) [4]. Furthermore, cardiac troponin I (cTnI) and N-terminal pro B-type natriuretic peptide (NT-proBNP) [5] also offer prognostic information. Nevertheless, there is a constant need for new biomarkers to predict the survival of patients, suffering from this multifaceted disease.

MicroRNAs (miRNAs, miRs) stand as potential novel prognostic and diagnostic biomarkers for various pathologies, including ACS. MiRNAs are small, non-coding RNAs that regulate the epigenetic processes, which then affect cell function [6]. A previous study indicated that miR-223 may be a predictor of mortality in patients with symptomatic coronary artery disease (CAD) [7]; however, studies have presented contradictory results regarding low or high expression of miR-223 as an independent predictor of major adverse cardiovascular events (MACE) and cardiovascular death in patients suffering from CAD [7,8,9]. Additionally, miR-125a was also found to play a part in cardiovascular diseases [10], for instance, as a cell survival stimulator and apoptosis inhibitor in cardiomyocytes [11]. In our previous preliminary observation, remarkable modulation of miR-223, as well as of miR-125, was detected in a patient population with high on-treatment platelet aggregation levels as opposed to in antiplatelet-responsive patients after acute myocardial infarction [12]. Consequently, the aim of the present study was to investigate whether miR-223 bears the prognostic capacity to predict long-term all-cause mortality in patients with NSTE-ACS. Furthermore, we evaluated the association between miR-125a, miR-223 and diabetes mellitus, as well as possible correlations with HbA1c levels.

## 2. Materials and Methods

### 2.1. Study Design

This study followed a prospective observational study design, and was performed between July 2012 and January 2020 at the Medical University of Vienna. The active recruitment period for the trial was between July 2012 and mid-June 2015. Follow-up was then conducted until January 2020. The study’s protocol was in accordance with the Declaration of Helsinki and was approved by the Ethics Committee of the Medical University of Vienna (approval number: 1051/2012, date of approval 22 April 2012). The study included consecutive patients, who suffered from ACS and had been referred for percutaneous coronary intervention (PCI). Expression of miR-125a and miR-223 was then assessed in all 109 study participants, who suffered from NSTE-ACS. Patients had to be at least 18 years of age, had to be able to provide written informed consent before study entry and had to receive planned treatment with a potent P2Y_12_ inhibitor. Notably, miRNAs were only measured in patients on ticagrelor/prasugrel to prevent bias, since platelets are the main source of circulating miRNAs [13]. The only exclusion criterion was any participation in interventional trials. Data regarding long-term survival were acquired through queries of the Austrian death registry until January 2020.

### 2.2. Study Endpoints

Long-term all-cause mortality was considered as the primary endpoint of the study. The secondary endpoint was MACE within the first year after discharge. In this context, MACE was defined as nonfatal myocardial infarction, nonfatal stroke and cardiovascular death. Determination of the composite endpoint followed the current universal criteria [14,15]. The safety endpoint was defined as thrombolysis for acute myocardial infarction (TIMI; minimal/minor/major) bleeding. Further, we aimed to investigate an association between miR-223, miR-125a and HbA1c.

### 2.3. RNA Preparation and Detection and MiRNA Quantification by Applying Quantitative PCR

Blood plasma RNA was extracted by the miRVANA PARIS Kit (Invitrogen, Waltham, MA, USA). Further, isolated RNA was reverse-transcribed by using the TaqMan miRNA Reverse Transcription kit (Applied Biosystems, Waltham, MA, USA) in accordance with the manufacturer’s protocol. MiRNA expression was detected by real time quantitative polymerase chain reaction (qRT-PCR). TaqMan miRNA Assay kits (Applied Biosystems) were then employed for the corresponding miRNAs on the CFX384 Touch Real-Time PCR Detection System (BioRad Inc., Hercules, CA, USA). Next, cel-miR-39 was spiked in an exogenous normalizer. Reactions were run in triplicate, and the mean values were then used for all analyses to control the variability associated with methodological factors. MiRNA levels are expressed as 2-ΔCT (miRNA-cel-miR-39) [6,16].

### 2.4. Statistical Analysis

The following data are presented as mean ± standard deviation (SD), median and interquartile range (IQR; defined as the range from the 25th to the 75th percentile), 95% confidence interval (CI), numbers (*n*) and percentages (%), as applicable. MiR-125a and miR-223 values were converted into decadic logarithms (log10) to homogenize skewed data. A prior evaluation of statistical power was not performed due to the explorative nature of the study. The Mann–Whitney U-test and the X^2^-test were used for comparisons between groups. Receiver operating characteristic (ROC) analysis was performed for determination of miR-223s’ and miR-125as’ ability to predict long-term all-cause mortality. For survival analyses, Kaplan–Meier curves and the Mantel–Cox regression test were applied. A multivariate Cox-regression analysis was conducted to establish independent parameters influencing long-term all-cause mortality. As only a limited number of events were determined, only four parameters were entered into the equation to prevent overfitting of the model. In this regard, the following variables were included: high expression of miR-223 (>7.1), age ≥ 65 years, diabetes mellitus and dyslipidemia. For calculation of the bivariate correlations between metric variables, Spearman’s rho was applied. Statistical analyses were conducted using commercially available statistical software (IBM SPSS Statistics 25, IBM, Armonk, NY, USA).

### 2.5. Group Stratification

For the investigation of miR-223s’ and miR-125as’ ability to predict long-term all-cause mortality, ROC analysis was applied. The optimal cut-off value of miR-223 was established by calculating the greatest sum of sensitivity and specificity of the ROC coordinate points, as previously described [17]. Subsequently, stratification of two groups based on a cut-off value of 7.1 was implemented, resulting in a low miR-223 (≤7.1) cohort and a high miR-223 (>7.1) cohort. Correspondingly, the optimal cut-off point for miR-125a was calculated (5.5).

## 3. Results

### 3.1. Patient Demographics

Detailed baseline characteristics, including concomitant medication, laboratory data, risk factors, past medical history and ACS data are summarized in Table 1. The majority of the study participants were male (73%), with a mean age of 61 years. Arterial hypertension was the most commonly observed concomitant disease, comprising 70%, followed by dyslipidemia at 57%. Diabetes mellitus occurred in 26% of the participants. About 65% reported smoking, whereas 46% disclosed a family history of coronary artery disease (CAD). The whole study cohort (100%) received aspirin after discharge. Additionally, ticagrelor and prasugrel were administered to 86% and 11% of participants, respectively. However, in 4%, a switch from potent platelet inhibitors to clopidogrel was observed throughout the hospital stay. Other frequently administered drugs included statins (91%), β-blockers (90%) and angiotensin converting enzyme (ACE) inhibitors/angiotensin II receptor blockers (ARBs; 88%).

### 3.2. Association of MiR-223 with Long-Term All-Cause Mortality

In accordance with the cut-off value of miR-223 (7.1), which was acquired by the ROC analysis, the study population (*n* = 109) was separated into two subgroups. The low miR-223 subgroup (*n* = 50) had miR-223 levels < 7.1 (6.3 ± 0.8), whereas the high miR-223 cohort (*n* = 59) presented with miR-223 plasma expression above 7.1 (7.8 ± 0.6; *p* < 0.001).

**Figure 1:**
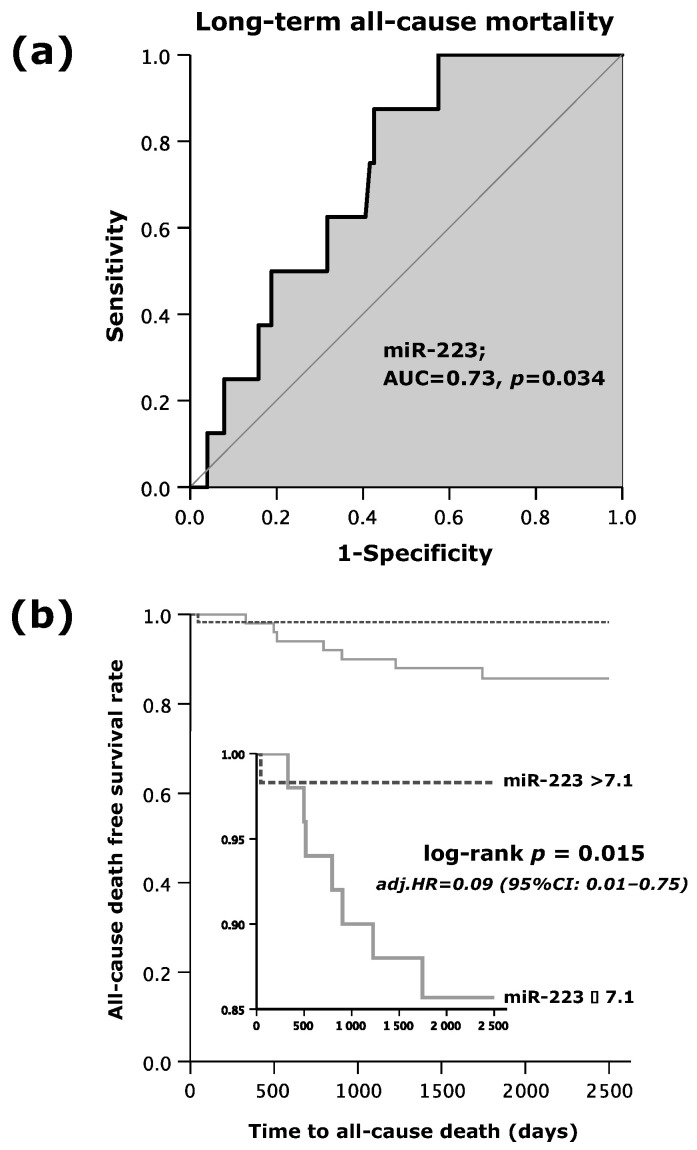
(**a**) Receiver operating curve (ROC) analysis for microRNA (miR)-223 to predict long-term all-cause mortality; and (**b**) Kaplan–Meier survival analysis for long-term all-cause mortality regarding high or low values of miR-223.

As shown by Figure 1a, the area under the curve (AUC = c-index) was 0.73 (95%CI: 0.58–0.86; *p* = 0.034). MiR-223s’ sensitivity in the prediction of all-cause mortality within the follow-up period of 7.5 years was 88%, and specificity made up 57%. The positive predictive value reached 14%, whereas the negative predictive value extended to 98%. Accordingly, the positive likelihood ratio and negative likelihood ratio were 2.1 and 0.2, respectively (Table 2).

### 3.3. Patients’ Characteristics According to Low and High MiR-223 Expression

In general, homogeneous frequencies regarding data on risk factors, past medical history, laboratory parameters, concomitant medication and information regarding the event were observed (Table 1). However, arterial hypertension occurred more often in patients with high expression levels of miR-223 (78%) as compared to those with lower levels of miR-223 (60%; *p* = 0.042). Similarly, in the high miR-223 subgroup, participants suffered more frequently from multivessel disease (33% vs. 14%; *p* = 0.020) and chronic obstructive pulmonary disease (COPD; 9% vs. 0%; *p* = 0.035) than the low miR-223 cohort. Regarding laboratory data, miR-125a was also higher in patients with high miR-223 expression than in patients with low miR-223 expression (5.3 ± 0.7 vs. 4.1 ± 1.0; *p* < 0.001). Likewise, total stent length was elevated in the high miR-223 subgroup as opposed to the low miR-223 one (39.3 ± 28.6 vs. 24.4 ± 15.8; *p* = 0.011).

### 3.4. Survival Analysis According to MiR-223 and MiR-125a

Altogether, 8 out of 109 patients (7%) experienced the primary endpoint of long-term all-cause mortality. In all 59 patients with miR-223 levels beyond 7.1, only 1 patient (2%) died. However, of the 50 patients with lower miR-223 expression (≤7.1), 7 patients (14%) passed away, indicating that higher values of miR-223 (>7.1) were associated with a favorable outcome (Table 3). As shown in Figure 1b, the time to event analysis demonstrated a 7-fold increased death rate in patients with low miR-223 expression (log rank test *p* = 0.015) as opposed to those with increased miR-223 expression (>7.1). A multivariate Cox regression analysis showed that miR-223 emerged as an independent predictor for long-term all-cause mortality.

Patients in the low miR-223 subgroup were at a 91% increased hazard of death within the follow-up period of 7.5 years as compared to those who presented with miR-223 > 7.1 (adjusted (adj.) hazard ratio (HR) = 0.09; 95%CI: 0.01–0.75; *p* = 0.026; Table 4). Apart from the expression of circulating miR-223, no other independent predictors for long-term all-cause death were determined by the Cox regression analysis.

The expression of circulating miR-125a was not predictive of long-term all-cause mortality (AUC = 0.62; 95%CI: 0.44–0.80; *p* = 0.256).

### 3.5. MACE and TIMI Bleeding Events According to MiR-223 Expression

Regarding the secondary endpoint (MACE) and the safety endpoint (minimal/minor/major TIMI bleeding) no remarkable differences between the subgroups were found (*p* = 0.696 and *p* = 0.509, respectively).

### 3.6. Distribution of MiR-125a and MiR-223 in Regard to Diabetes Mellitus

**Figure 2:**
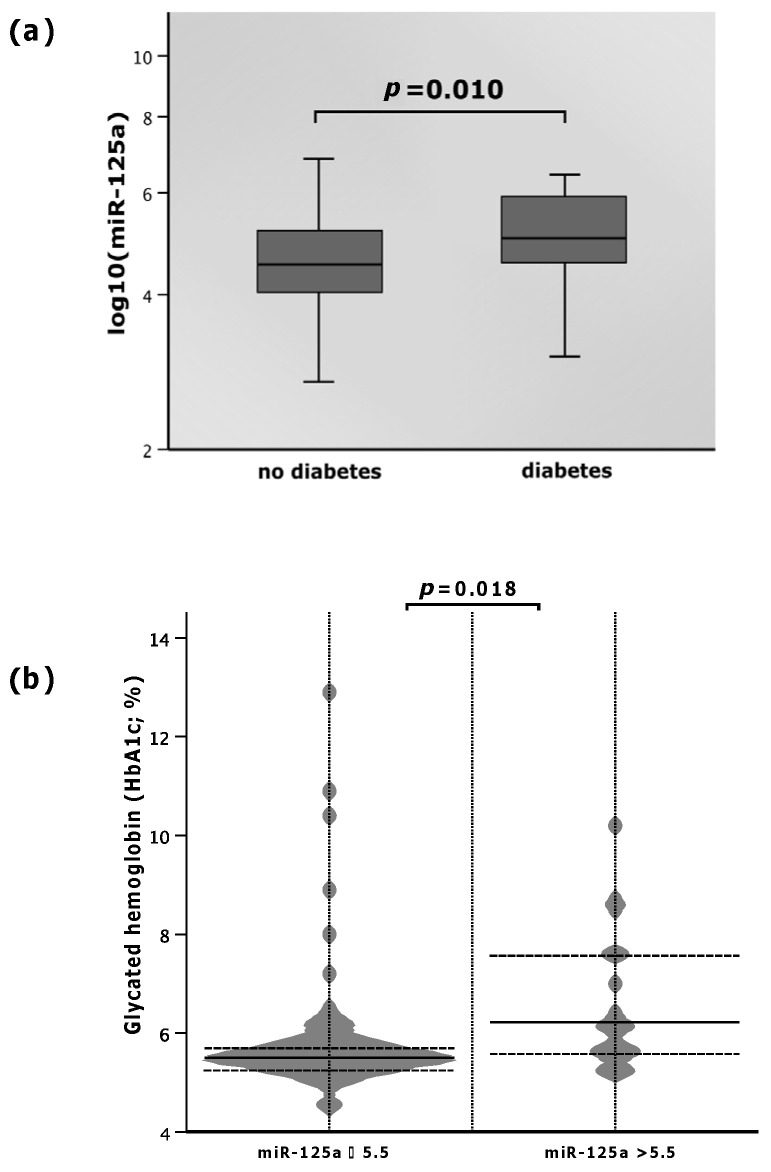
(**a**) MicroRNA (miR)-125a with regard to diabetes mellitus; (**b**) glycated hemoglobin (HbA1c) according to high and low values of miR-125a.

The levels of miR-125a varied with respect to the diagnosis of diabetes mellitus. Patients suffering from diabetes mellitus presented with elevated plasma miR-125a as opposed to those without the disease. As depicted in Figure 2, the median level of miR-125a in diabetics was 5.0 (IQR: 4.5–5.9), whereas non-diabetics showed a 10% lower median miR-125a value of 4.5 (IQR: 4.0–5.2, *p* = 0.010; Figure 2a).

No significant differences in the miR-223 expression in patients with vs. without diabetes mellitus were found (*p* = 0.063).

### 3.7. Association between MiR-125a, MiR-223 and HbA1c Levels

Patients within the study cohort showed an association between miR-125a and HbA1c. When miR-125a was ≤5.5, HbA1c was 9.8% lower (median: 5.5%; IQR: 5.3–5.9%) than that with elevated miR-125a levels (>5.5) (median: 6.1%; IQR: 5.6–7.6%; *p* = 0.018; Figure 2b). Spearman’s rho obtained 0.211, but did not reach statistical significance (*p* = 0.069).

No association between low or high miR-223 expression (≤7.1; >7.1) and HbA1c was found (*p* = 0.376).

## 4. Discussion

The central findings of the present study, investigating the expression of miR-223 and miR-125a in patients with diagnosed NSTE-ACS, were as follows. Firstly, elevated expression of miR-223 was associated with a favorable long-term outcome. Secondly, patients suffering from diabetes mellitus presented with an elevation in plasma miR-125a expression as opposed to those without the disease. Thirdly, increased miR-125a levels were linked to an elevated HbA1c concentration.

There is growing evidence that ACS is associated with distinct changes in miRNA expression [18]. The myocardial damage occurring in infarcted hearts is followed by apoptosis, inflammation and fibrosis of the cardiac tissue. However, the mechanism underlying the pathophysiology of the myocardial infarction on a molecular level is not yet fully elucidated. Our study is the first to provide evidence that the expression of miR-223 differs between patients diagnosed with NSTE-ACS, suggesting its involvement in the adaptation of the cardiovascular system after an infarction. In our study, higher miR-223 expression was found to have a predictive value for favorable outcome regarding the long-term all-cause mortality within the 7.5-year follow-up period. However, the influence of miR-223 on the cardiovascular system remains conflicting. Some authors have shown deleterious effects of increased miR-223, including enhancement of fibrosis [19] and stimulation of atherogenesis [20]. In contrast, other authors reported beneficial effects of elevated miR-223 expression, including protection against ischemia–reperfusion injury [21,22] and apoptosis, as well as myocarditis [23]. In a murine model, miR-223 ameliorated fibrosis, inflammation and apoptosis, and mediated angiogenesis [24]. MiR-223 even decreased tissue factor expression and procoagulant activity, implying its potential protective function following endothelial injury [25]. Hence, the increase in the expression of miR-223 could either be a protective mechanism against an acute cardiovascular event in that patient population or may contribute to cardiovascular injury. In our observation, higher expression of miR-223 was linked to lower mortality and a more favorable outcome. Recently, another study has also demonstrated elevated miR-223 to be linked to good prognoses in patients with breast cancer [26], as well as in patients with aortic stenosis undergoing transcatheter aortic valve implantation [27]. A relationship between improved survival and amplified miR-223 expression was further observed in patients suffering from sepsis. In this study, miR-223 was also measured in healthy controls, and their levels were significantly lower as compared to patients who suffered from sepsis (*p* < 0.001), whereas sepsis survivors had even higher miR-223 values than non-survivors (*p* = 0.001). These results indicate a protective function of increased miR-223 expression during sepsis [28]. In addition, this is supported by another study which demonstrated lower miR-223 levels in healthy controls [29]. However, data on miR-223 is also conflicting in healthy controls, since there is evidence that miR-223 values are amplified in this very population [18]. For example, another study reported on reduced miR-223 expression in patients with acute myocardial infarction as compared to patients without the disease (*p* < 0.005) [30].

In contrast, however, other studies have shown an increase in miR-223 expression to be associated with worse outcomes in patients who suffered from ACS [7,31,32,33]. One possible explanation for these conflicting results may be the different patient populations. We explicitly focused on patients who suffered from NSTE-ACS, in contrast to Schulte and colleagues [7], Elbaz et al. [31] and Hromadka and associates [33], who investigated miR-223 in all subtypes of ACS, or Scărlătescu and her team [32], whose patient collective solely comprised subjects diagnosed with STEMI. Notably, the latter two studies further confirmed reduced expression of miR-223 in a healthy control subpopulation [31,32]. In our preliminary results, we already demonstrated that miR-223 values differed between patients with STEMI and NSTE-ACS. This could be partly explained by the different atherosclerotic burdens of both conditions [6]. Since miR-223 differs between STEMI and NSTE-ACS, there is also a certain possibility that miR-223 has different associations with mortality among these patients.

Nevertheless, in our study, concomitant diseases, such as arterial hypertension, multivessel disease and COPD, occurred significantly more often in patients with increased miR-223 levels. An association between elevated circulating miR-223 and patients with COPD [34] and multivessel disease [6] has already been demonstrated. However, no studies reporting on increased levels of circulating miR-223 and arterial hypertension have been published yet. The versatile results on the association between miR-223, cardiovascular diseases and survival remain complex, and the contradictory findings require further investigation.

Another major observation in our study was the elevation of miR-125a in NSTE-ACS patients who had been diagnosed with diabetes mellitus as compared to non-diabetic patients. Additionally, we observed that low expression of miR-125 was accompanied by lower HbA1c concentrations. Data regarding the association between diabetes mellitus and miR-125a are rare; however, a few studies have already reported on the link between this versatile disease and upregulated miR-125a levels [35]. According to a study which investigated regulatory T cells in patients with and without type 1 diabetes mellitus (T1DM), miR-125a was significantly increased in those with T1DM as opposed to the non-diabetic control group. However, miR-125a was only overexpressed in T cells derived from pancreatic lymph nodes, and not in those from peripheral blood. This is in contrast to our findings, where miR-125a was obtained from blood plasma [36]. Further, our study not only included patients with T1DM, but also those with type 2 diabetes mellitus (T2DM). Analyses conducted in rodents have also confirmed enhanced miR-125a production in a model of T2DM [37]. Similar to our investigation, ACS patients with hyperglycemic fasting blood showed increased levels of plasma miR-125a as compared to those with a normoglycemic state under the same circumstances [38]. In this context, renoprotective properties of upregulated miR-125a were suggested [39,40].

In summary, we want to emphasize the high negative predictive value (98%) of miR-223 for long-term all-cause mortality. Patients with miR-223 expression above 7.1 at baseline had favorable 7.5-year survival. Furthermore, we wish to highlight the association between increases in miR-125a expression and diabetes mellitus, as well as its diagnostic parameter, HbA1c.

### Limitations

Our study has two major limitations. Firstly, there may be bias due to the observational study design, which did not include a healthy control group. Due to this, there remains a likelihood that our results were also influenced by other factors. Secondly, this study investigated a rather small sample size of 109 participants. However, this number of patients is similar to that in other studies which explored miRNAs and survival outcomes. Further, we did not perform any miRNA profiling/screening in our analysis, and miRNA sequencing may, possibly, have enabled us to determine novel miRNAs with higher predictive values for our secondary endpoints. Altogether, the present results should be confirmed in a larger, preferably multi-centric study by using RNA-sequencing technology before the respective miRNAs can be used in risk stratification or as a prediction factor in clinical practice.

## 5. Conclusions

Our present study demonstrates the predictive value of miR-223 regarding long-term survival in NSTE-ACS patients. Additionally, miR-125a expression was found to be higher in patients suffering from diabetes mellitus. Importantly, further studies are needed to elaborate upon the role of these miRNAs in the pathogenesis and prognosis of patients with NSTE-ACS.

## Figures and Tables

**Table 1:** Patient demographics.

Patient Demographics	Overall *n* = 109 (100)	miR-223 ≤ 7.1 *n* = 50 (46)	miR-223 > 7.1 *n* = 59 (54)	*p*-Value
miR-223	7.1 ± 1.1	6.3 ± 0.8	7.8 ± 0.6	**<0.001**
Age (years), mean ± SD	60.6 ± 12.6	60.3 ± 12.1	60.9 ± 13.1	0.927
Sex (male), *n* (%)	80 (73)	38 (76)	42 (71)	0.571
**Risk factors/past medical history *n* (%)**				
Body mass index	27.8 ± 5.1	27.5 ± 5.0	28.1 ± 5.1	0.415
Arterial hypertension	76 (70)	30 (60)	46 (78)	**0.042**
Multivessel disease	26 (24)	7 (14)	19 (33)	**0.020**
Dyslipidemia	62 (57)	30 (60)	32 (54)	0.545
Diabetes mellitus	28 (26)	10 (20)	18 (31)	0.211
Peripheral artery disease	6 (6)	1 (2)	5 (9)	0.140
Cerebrovascular disease	5 (5)	3 (6)	2 (3)	0.516
Chronic obstructive pulmonary disease	5 (5)	0 (0)	5 (9)	**0.035**
Smoking	71 (65)	35 (70)	36 (61)	0.327
Family history of CAD	50 (46)	20 (40)	30 (51)	0.257
Prior myocardial infarction	25 (23)	14 (28)	11 (19)	0.247
Prior PCI	12 (11)	5 (10)	7 (12)	0.757
**Laboratory data (mean ± SD)**				
White blood cell count (×10^9^/L)	9.8 ± 3.3	10.1 ± 3.5	9.6 ± 3.2	0.593
Platelets (×10^9^/L)	249.7 ± 77.8	247.5 ± 77.1	251.5 ± 79.0	0.874
Hemoglobin (g/dL)	13.8 ± 1.8	13.9 ± 1.7	13.6 ± 1.8	0.444
C-reactive protein (mg/dL)	2.4 ± 3.1	2.6 ± 3.3	2.1 ± 3.0	0.354
Fibrinogen (mg/dL)	409.1 ± 106.4	395.0 ± 108.2	421.3 ± 104.3	0.139
Creatinine (mg/dL)	1.0 ± 0.5	1.1 ± 0.6	1.0 ± 0.3	0.896
Troponin T (µg/L)	0.3 ± 0.7	0.4 ± 0.9	0.2 ± 0.4	0.251
HbA1c (%)	6.1 ± 1.5	6.0 ± 1.6	6.2 ± 1.4	0.376
GFR (ml/min/1.73 m^2^)	78.1 ± 22.5	78.4 ± 24.7	77.8 ± 20.7	0.752
miR-125a	4.7 ± 1.7	4.1 ± 1.0	5.3 ± 0.7	**<0.001**
**Concomitant medications *n* (%)**				
Aspirin	109 (100)	50 (100)	59 (100)	
Clopidogrel	4 (4)	3 (6)	1 (2)	0.238
Ticagrelor	89 (86)	40 (83)	49 (88)	0.547
Prasugrel	11 (11)	5 (10)	6 (11)	0.961
ß-blockers	96 (90)	42 (86)	54 (93)	0.210
Angiotensin-converting enzyme (ACE) inhibitors/Angiotensin II receptor blockers (ARB)	94 (88)	42 (86)	52 (90)	0.534
Calcium channel blockers	20 (19)	12 (25)	8 (14)	0.157
Proton pump inhibitors	85 (79)	41 (84)	44 (76)	0.319
Statins	97 (91)	43 (88)	54 (93)	0.344
Antidiabetic drugs	19 (18)	7 (14)	12 (21)	0.388
**ACS data**				
Number of stents per patient	1.4 ± 1.1	1.1 ± 0.8	1.6 ± 1.3	0.213
Total stent length	32.3 ± 24.5	24.4 ± 15.8	39.3 ± 28.6	**0.011**

Data are reported as mean ± standard deviation (SD), *n* (number of patients) or percentages; miR, microRNA; CAD, coronary artery disease; HbA1c, glycated hemoglobin; GFR, glomerular filtration rate; PCI, percutaneous coronary intervention, STEMI, ST-segment elevation myocardial infarction; NSTE-ACS, non-ST-segment elevation acute coronary syndrome. Bold *p*-values indicate statistical significance.

**Table 2:** Statistical estimates for the prediction of long-term all-cause mortality depending on miR-223 levels.

Long-Term All-Cause Mortality *n* = 8 (7.3%)									
	Test								
	c-Index (95%CI)	*p*-Value	Cut-Off Value	Sensitivity, %	Specificity, %	Positive Predictive Value, %	Negative Predictive Value, %	LR+	LR−
low miR-223 vs. high miR-223	0.73 (0.58–0.86)	**0.034**	7.1	88	57	14	98	2.1	0.2

NSTE-ACS, non-ST-segment elevation acute coronary syndrome; miR, microRNA; 95%CI = 95% confidence interval; LR+, positive likelihood ratio; LR−, negative likelihood ratio. Bold *p*-values indicate statistical significance.

**Table 3:** Event data.

Event	Overall *n* = 109 (100)	miR-223 ≤ 7.1 *n* = 50 (46)	miR-223 > 7.1 *n* = 59 (54)	*p*-Value
*Long-term all-cause mortality*	8 (7)	7 (14)	1 (2)	**0.014**
*MACE (1 year)*	10 (9)	4 (8)	6 (10)	0.696
*Minimal/minor/major TIMI bleeding (1 year)*	54 (50)	26 (55)	28 (49)	0.509

miR, microRNA; MACE, major adverse cardiac event; TIMI, thrombolysis in myocardial infarction. Bold *p*-values indicate statistical significance.

**Table 4:** Multivariate Cox regression model for prediction of long-term all-cause mortality.

Variable	HR	95%CI		*p*-Value
		Lower	Upper	
*miR-223 > 7.1*	0.09	0.01	0.75	**0.026**
*Age ≥ 65 years*	1.62	0.39	6.81	0.509
*Diabetes mellitus*	2.92	0.63	13.51	0.170
*Dyslipidemia*	0.30	0.07	1.32	0.111

HR, hazard ratio; 95%CI, 95% confidence interval; miR, microRNA. Bold *p*-values indicate statistical significance.

## Data Availability

The data presented in this study are available on request from the corresponding author. The data are not publicly available due to privacy restrictions.
